# Triple therapy versus sequential therapy for the first-line *Helicobacter pylori* eradication

**DOI:** 10.1186/s12876-017-0579-8

**Published:** 2017-01-21

**Authors:** Ji Young Chang, Ki-Nam Shim, Chung Hyun Tae, Ko Eun Lee, Jihyun Lee, Kang Hoon Lee, Chang Mo Moon, Seong-Eun Kim, Hye-Kyung Jung, Sung-Ae Jung

**Affiliations:** Department of Internal Medicine, Ewha Womans University School of Medicine, Ewha Medical Research Institute, 1071 Anyangcheon-ro, Yangcheon-gu, Seoul, 158-710 South Korea

**Keywords:** *Helicobacter pylori*, Anti-bacterial agents, First-line triple therapy, Sequential therapy

## Abstract

**Background:**

The eradication rate of *Helicobacter pylori (H. pylori)* with triple therapy which was considered as standard first-line treatment has decreased to 70–85%. The aim of this study is to compare 7-day triple therapy versus 10-day sequential therapy as the first line treatment.

**Methods:**

Data of 1240 *H. pylori* positive patients treated with triple therapy or sequential therapy from January 2013 to December 2015 were analyzed retrospectively. The patients who had undertaken previous *H. pylori* eradication therapy or gastric surgery were excluded.

**Results:**

There were 872 (74.3%) patients in the triple therapy group, and 302 (25.7%) patients in the sequential therapy group. There was no significant difference between the two groups regarding age, residence, comorbidities or drug compliance, but several differences were noted in endoscopic characteristics and indication for the treatment. The eradication rate of *H. pylori* by intention to treat analysis was 64.3% in the triple therapy group, and 81.9% in the sequential therapy group (*P* = 0.001). In per protocol analysis, *H. pylori* eradication rate in the triple therapy and sequential therapy group was 81.9 and 90.3%, respectively (*P* = 0.002). There was no significant difference in overall adverse events between the two groups (*P* = 0.706). For the rescue therapy, bismuth-containing quadruple therapy showed comparable treatment efficacy after sequential therapy, as following triple therapy.

**Conclusions:**

The eradication rate of triple therapy was below the recommended threshold. Sequential therapy could be effective and tolerable candidate for the first-line *H. pylori* eradication therapy*.*

## Background

The prevalence of *Helicobacter pylori (H. pylori)* infection has decreased over the past decade, changed from 66.9 to 54.4% between 1998 and 2011, but its prevalence is still high in Korea [1]. *H. pylori* infection is a known risk factor of upper gastrointestinal diseases, such as chronic gastritis, peptic ulcer disease, mucosa-associated lymphoid tissue (MALT) lymphoma, and gastric cancer [2, 3]. Eradication of *H. pylori* reduces the recurrence rate of peptic ulcer disease or recurrent gastric cancer after endoscopic resection of early gastric cancer, and it also induces the remission of MALT lymphoma [4–6]. Therefore, *H. pylori* eradication has critical role in promoting national health in Korea, where 95% of confirmed *H. pylori* strains have highly virulent East Asian-type cytotoxin-associated gene A which is potent in causing gastric cancer [7, 8].

Triple therapy (TT) consists of proton-pump inhibitor (PPI), clarithromycin, and amoxicillin has been considered as standard first-line treatment for *H. pylori* in Korea since 1998 [9]. Recently updated Korean guideline also recommended TT as the first-line regimen [10]. However, the efficacy of TT has decreased progressively. The recent nationwide survey reported the decreasing trend of eradication rate of TT which was 84.9–87.5% from 2001 to 2007, but 80.0–81.4% from 2008 to 2010 (*P* < 0.0001) [11]. The most important factor of reduced efficacy of TT is increasing antibiotic resistance of *H. pylori*, especially to clarithromycin [12]. The primary resistance rate to clarithromycin increased from 23.7 to 71.2%, whereas amoxicillin increased from 6.3 to 14.9% during the period of 2003–2012 [13].

Therefore, several protocols have been suggested in order to overcome treatment failure of TT, including the extending of treatment duration, the use of four-drug regimen such as sequential therapy (SET), concomitant therapy, hybrid therapy, and the prescription of novel antibiotics such as levofloxacin [14]. Reasonable treatment regimens need to attain *H. pylori* eradication rate of higher than 80.0% by intention to treat (ITT) analysis, and higher than 90.0% by per protocol (PP) analysis [15, 16]. Several previous meta-analyses reported the superiority of SET than TT [17, 18], whereas other studies revealed conflicting results [19, 20].

In Ewha Womans University Medical Center, SET has been tried as an alternative first-line treatment *for H. pylori* since 2013. So, we aimed to compare 7-day TT with 10-day SET as the first line treatment in our medical center. We also evaluated the adverse events of the two regimens, clinical factors associated with successful eradication, and effectiveness of the second line treatment after these two treatments.

## Methods

### Study subjects

From January 2013 to December 2015, 1240 patients who were older than 18-year old, diagnosed with *H. pylori* infection and treated with TT or SET at Ewha Womans University Hospital were enrolled retrospectively. *H. pylori* infection was confirmed by histology, rapid urease test (HP Kit™, Jongkeundang, Korea), C-urea breath test or serum *H. pylori* anti-body test. At least 4 weeks after treatment, *H. pylori* eradication was demonstrated by any of these tests. The patients who had undertaken previous *H. pylori* eradication therapy or gastric surgery were excluded.

We evaluated demographic information, residence area, current status of smoking and alcohol consumption, comorbidities, endoscopic diagnosis, indication for *H. pylori* eradication, drug compliance, and treatment-related adverse events through medical records review. Endoscopic findings and the results of endoscopic biopsies were also reviewed retrospectively. For detailed analysis, drug compliance was divided into two categories; good or poor compliance. Good compliance was defined if the patient took more than 80% of the prescribed medicine, and who took less than 80% of prescribed medicine was belonged to poor compliance group. For the PP analysis, patients who were poorly compliant or lost to follow-up were excluded.

Standard TT for seven days consists of twice a day amoxicillin (1000 mg), clarithromycin (500 mg), and standard dose of PPI. SET for 10 days consists of twice a day amoxicillin (1000 mg), standard dose of PPI for 5 days, followed by twice a day clarithromycin (500 mg), metronidazole (500 mg), and standard dose of PPI for another 5 days.

This study was approved by the Institutional Review Board of our medical center (IRB number; 2016-04-051-002).

### Statistical analyses

All statistical analyses were performed with using SPSS program, version 22.0. Continuous variables were reported as the mean with the standard deviation. To analyze the baseline clinical characteristics, adverse events and eradication rates between the two groups, Student *t-*test or the Mann-Whitney *U* test was used for continuous variables, and the chi-square or the Fisher’s exact test was used for categorical variables. *H. pylori* eradication rates were demonstrated by ITT and PP analyses. Univariate and multivariate logistic regression were performed for evaluating independent associated factors with successful *H. pylori* eradication. The *P* value of < 0.05 was considered as statistical significance.

## Results

### Baseline characteristics

**Fig. 1:**
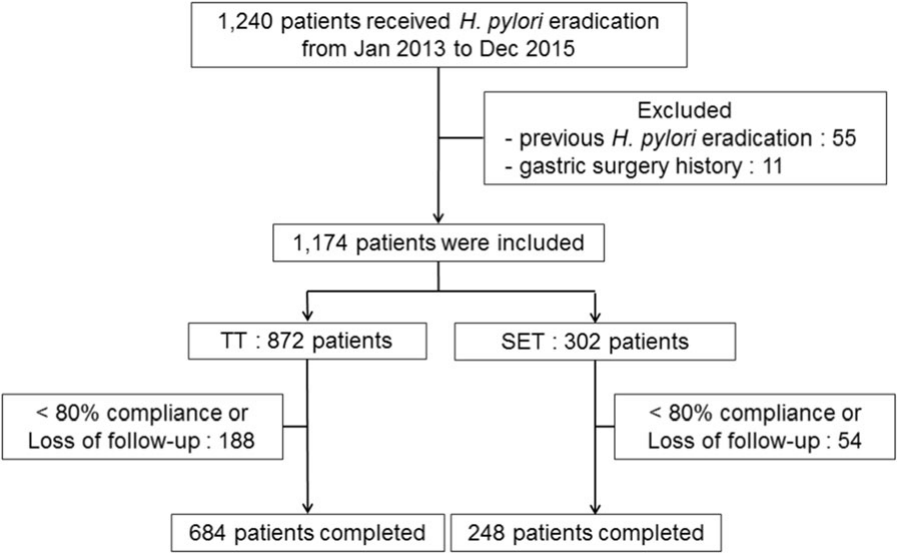
The flowchart of enrolled patients. *H. pylori Helicobacter pylori*, Jan January, Dec December, TT triple therapy, SET sequential therapy

A total of 1240 patients received *H. pylori* eradication therapy from January 2013 to December 2015. After excluding 66 patients who had previous *H. pylori* eradication or gastric surgery, 1174 patients were included finally. There were 872 patients in the TT group and 302 patients in the SET group. A detailed flowchart of the enrolled patients is shown in Fig. 1. The baseline characteristics of the study population are summarized in Table 1. There was no significant difference between the two groups regarding age, residence, comorbidities or drug compliance. But, more males and more patients who were diagnosed with *H. pylori* infection by histology were included in the SET group than the TT group. Several differences were found in the endoscopic characteristics - atrophy or metaplastic gastritis (*P* = 0.009), and gastric ulcers (*P* = 0.011) were more prevalent in the SET group, whereas chronic gastritis (*P* < 0.001) and duodenal ulcers (*P* = 0.008) were more prevalent in the TT group. In terms of indication of *H. pylori* eradication, significantly higher portion of patients received SET after endoscopic resection of gastric neoplasms such as early gastric cancers (*P* < 0.001) or gastric adenomas (*P* = 0.009).

**Table 1:** Baseline clinical characteristics

	TT	SET	*P* value
	*N* = 872 (74.3%)	*N* = 302 (25.7%)	
Age (mean ± SD)	52.26 ± 13.52	51.83 ± 12.17	0.607
Male	477 (54.7)	192 (63.6)	0.007
Residence			0.510
Seoul	695 (79.7)	246 (81.5)	
Another area	177 (20.3)	56 (18.5)	
Smoking	217 (24.9)	113 (37.4)	<0.001
Alcohol	389 (41.3)	91 (39.2)	<0.001
Diabetes mellitus	98 (11.2)	33 (10.9)	0.882
Hypertension	185 (21.2)	71 (23.5)	0.405
Chronic kidney disease	12 (1.4)	4 (1.3)	0.999
Chronic liver disease	24 (2.8)	7 (2.3)	0.685
Ischemic heart disease	23 (2.6)	12 (4.0)	0.239
Compliance > 80%	707 (81.1)	256 (84.8)	0.150
*H. pylori* test			<0.001
Histology	293 (33.6)	182 (60.3)	
Rapid urease test	586 (64.9)	116 (38.4)	
Urea breath test	8 (0.9)	2 (0.7)	
Serology	5 (0.6)	2 (0.7)	
Endoscopic diagnosis			
Chronic gastritis	201 (23.1)	31 (10.3)	<0.001
Atrophy or metaplasia	332 (38.1)	141 (46.7)	0.009
Gastric ulcer	274 (31.4)	119 (39.4)	0.011
Duodenal ulcer	331 (38.0)	89 (29.5)	0.008
Duodenitis	52 (6.0)	18 (6.0)	0.998
Indication of *H. pylori* eradication			
Peptic ulcer disease	522 (59.9)	148 (49.0)	0.001
Endoscopic resection of EGC	21 (2.4)	24 (7.9)	<0.001
Endoscopic resection of adenoma	36 (4.1)	24 (7.9)	0.009
MALT lymphoma	4 (0.5)	2 (0.7)	0.651
*H. pylori* gastritis	83 (9.5)	23 (7.6)	0.320
Atrophy or metaplasia	130 (14.9)	61 (20.2)	0.032

*TT* triple therapy, *SET* sequential therapy, *SD* standard deviation, *H. pylori Helicobacter pylori, EGC early gastric cancer, MALT mucosa associated lymphoid tissue*

### *H. pylori* eradication rates

**Fig. 2:**
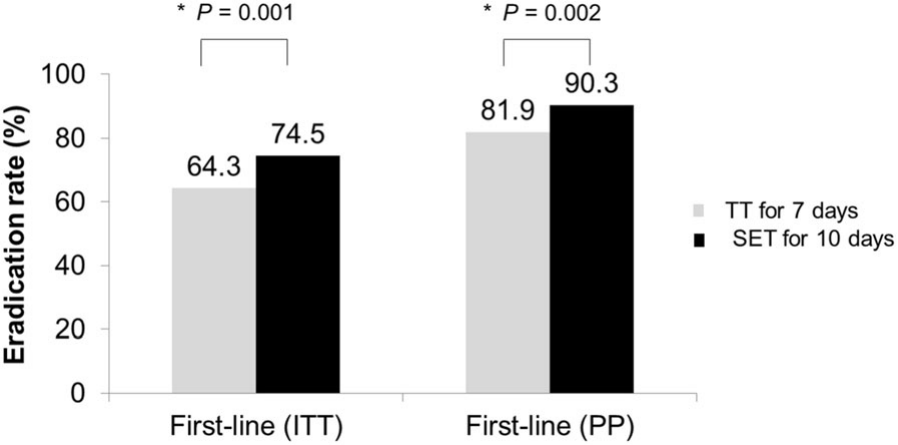
Comparison of eradication rate of *Helicobacter pylori* with first-line triple therapy with sequential therapy. The eradication rate of SET was significantly higher than TT by both ITT (*P* = 0.001) and PP (*P* = 0.002) analyses. TT triple therapy, SET sequential therapy, ITT intention to treat, PP per protocol

Among 872 patients receiving first-line TT, 684 (78.4%) patients completed the treatment with good compliance. In the SET group, 248 (82.1%) patients completed the treatment with good compliance in total of 302 patients. The eradication rate of SET was significantly higher than TT by both ITT (*P* = 0.001) and PP (*P* = 0.002) analyses. The TT showed the eradication rate of 64.3 and 81.9% by ITT and PP analyses, respectively. The overall eradication rate of SET was 81.9 in ITT, and 90.3% in PP analysis (Fig. 2).

### Clinical factors related to the *H. pylori* eradication

Possible clinical factors related to successful *H. pylori* eradication were also analyzed. But, there were no statistically significant factors to predict successful eradication in both univariate and multivariate analyses (Table 2).

**Table 2:** Clinical factors related to successful *Helicobacter pylori* eradication

	TT			SET		
	OR	95% CI	*P* value	OR	95% CI	*P* value
Univariate analyses						
Male gender	0.941	0.637 – 1.391	0.761	1.039	0.436 – 2.477	0.932
Age < 50 years	0.724	0.481 – 1.089	0.121	1.634	0.702 – 3.807	0.255
Residence – Seoul	0.819	0.509 – 1.320	0.413	1.154	0.375 – 3.553	0.803
Alcohol	0.942	0.628 – 1.415	0..775	0.909	0.391 – 2.115	0.824
Smoking	0.883	0.553 – 1.411	0.602	0.802	0.329 – 1.955	0.628
Diabetes mellitus	1.375	0.770 – 2.455	0.281	1.190	0.330 – 4.289	0.790
Hypertension	0.933	0.579 – 1.502	0.775	1.131	0.426 – 2.999	0.805
Indication						
Peptic ulcer disease	0.690	0.456 – 1.043	0.079	1.559	0.567 – 4.284	0.389
Malignant disease	0.647	0.297 – 1.410	0.273	1.604	0.461 – 5.581	0.458
Multivariate analysis						
Male gender	0.941	0.635 – 1.394	0.761	1.013	0.419 – 2.449	0.976
Age < 50 years	0.718	0.476 – 1.083	0.114	1.708	0.717 – 4.068	0.227
Residence – Seoul	0.792	0.490 – 1.282	0.343	1.203	0.280 – 3.831	0.750
Indication						
Peptic ulcer disease	0.679	0.447 – 1.030	0.069	1.538	0.554 – 4.272	0.409
Malignant disease	0.607	0.277 – 1.330	0.212	1.877	0.517 – 6.817	0.338

*TT* triple therapy, *SET* sequential therapy, *OR* odds ratio, *CI* confidence interval

### Comparison of treatment-related adverse events

During the treatment, 33 (3.8%) patients in the TT group, and 10 (3.3%) patients in the SET group had treatment-related adverse events. The most common adverse event was diarrhea (1.3% versus (vs.) 1.7%; TT vs. SET), followed by nausea or vomiting in both groups. But, there was no significant difference in the rate of specific adverse event as well as overall adverse events between the two groups (Table 3).

**Table 3:** Adverse events during first-line *Helicobacter pylori* eradication therapy

	TT	SET	*P* value
	*N* = 872 (74.3%)	*N* = 302 (25.7%)	
Total	33 (3.8)	10 (3.3)	0.706
Diarrhea	11 (1.3)	5 (1.7)	0.574
Nausea or vomiting	9 (1.0)	3 (1.0)	0.999
Abdominal pain	8 (0.9)	0 (0)	0.122
Skin rash	1 (0.1)	1 (0.3)	0.448
Metallic taste	1 (0.1)	0 (0)	0.999
Others^a^	5 (0.6)	2 (0.7)	0.999

*TT* triple therapy, *SET* sequential therapy, *H. pylori Helicobacter pylori*

^a^ Others included dyspepsia, bloating, and dizziness

### Second-line eradication therapy after first-line eradication failure

**Fig. 3:**
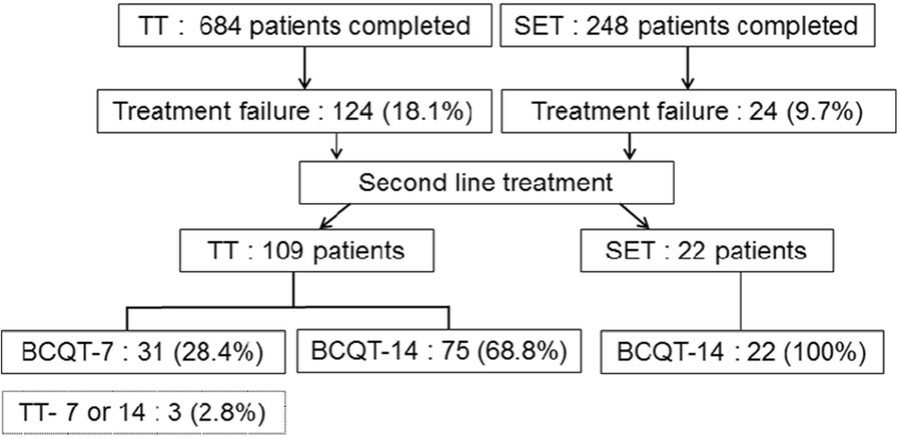
The flowchart of second-line treatment after failure of first-line eradication therapy TT triple therapy, SET sequential therapy, BCQT-7 bismuth-containing quadruple therapy for 7 days, BCQT-14 bismuth-containing quadruple therapy for 14 days

A detailed flow-chart of second-line eradication therapy after failure of first-line eradication therapy is shown in Fig. 3. Among 124 (18.1%) patients who failed in first-line TT, 109 patients received second-line eradication therapy; 28.4% for bismuth-containing quadruple therapy (BCQT) for 7 days (BCQT-7), 68.8% for BCQT for 14 days (BCQT-14), and 2.8% of patients for TT for 7 days. Data from the patients who received second-line TT could not be included for further analyses because of poor compliance or loss of follow-up. The eradication rate in patients who received BCQT-7 after failing first-line TT was 71.0 and 84.0% by ITT and PP analysis; eradication rate for BCQT-14 after TT was 85.3 and 95.5% by ITT and PP analysis, respectively. Twenty-four (9.7%) patients failed at their first-line SET, and 22 patients received BCQT-14 which showed the eradication rate of 72.7% in ITT and 84.2% in PP analysis. We found no significant differences in the overall eradication rates, compliance, adverse events between any of these three groups (Table 4).

**Table 4:** Comparisons of second-line treatment after failure of first-line eradication therapy

	TT → BCQT-7	TT → BCQT-14	SET → BCQT-14	*P* value
Eradication rate, % (*n*)				
ITT	71 (22/31)	85.3 (64/75)	72.7 (16/22)	0.162
PP	84 (21/25)	95.5 (64/67)	84.2 (16/19)	0.076
Compliance > 80%, % (*n*)	80.6 (25)	89.3 (67)	86.4 (19)	0.511
Adverse events, % (*n*)	25.8 (8)	22.7 (17)	18.2 (4)	0.839

*TT* triple therapy, *SET* sequential therapy, *BCQT-7* bismuth-containing quadruple therapy for 7 days, *BCQT-14* bismuth-containing quadruple therapy for 14 days, *ITT* intention to treat, *PP* per protocol

The most common complication after second-line treatment was nausea or vomiting in all of three groups. After failure of second-line eradication therapy in the SET group, two patients refused further treatment, and one patient received third-line eradication therapy consisted with twice a day standard dose of PPI, amoxicillin (1000 mg), and levofloxacin (500 mg) for 7 days. But, eradication of *H. pylori* also failed after third-line treatment.

## Discussion

Our study revealed that the eradication rate of TT was below the recommended threshold by both of ITT and PP analyses. This result is in accordance with the most recent meta-analysis for treatment of *H. pylori*, which concluded that SET was superior than TT showing the overall eradication rate of TT for 69.8 and 77.0%, and SET for 79.7 and 85.0% by ITT and PP analyses, respectively [14]. The most important cause of decreased efficacy of TT is considered as increasing antibiotic resistant rate, especially to clarithromycin [21]. The eradication rate of *H. pylori* was significantly different depending on the resistance or sensitivity to clarithromycin of the strain; 67.9% for clarithromycin-resistant strains and 95.5% for the clarithromycin-sensitive strains [12].

Previous studies proposed several clinical factors associated with *H. pylori* eradication failure including, age, gender, smoking, previous antibiotics usage [22, 23]. The most recent study in Korea reported that female gender could be associated with treatment failure, based on the fact that *H. pylori* strain with point mutation in the 23S rRNA were preferentially infected in women which could result in treatment failure with clarithromycin [24]. Also, smoking may increase treatment failure by reducing antibiotics delivery to gastric mucosa, because smoking decreases gastric blood flow and mucus secretion and smoking itself is an indicator for poor compliance [24–26]. However, we could not find any statistically significant clinical factor to predict successful eradication of *H. pylori.*

Our study supports SET as an alternative first-line treatment for several reasons. First, SET achieved reasonable target by both of ITT and PP analyses, whereas TT showed unacceptable efficacy. The reason for relatively higher efficacy of SET for *H. pylori* eradication compared with TT could be based on decreased resistance rate to metronidazole [27], because the resistance to nitroimidazole reduces the efficacy of sequential therapy up to 50% [21, 28, 29]. The resistance rate to metronidazole was reported 40.5% during 1994–1999 [30], 49.6% between 2003 and 2005 [31], and 27.5% between 2003 and 2009 [12] in Korea. And clarithromycin resistance is thought to have less effect on the efficacy of SET than on TT [32]. Second, treatment-related adverse events of SET were tolerable in most of the patients. There was no patient who discontinued the treatment due to treatment-related adverse event in our study. Also, no significant differences were found regarding overall complication rates or incidence of individual complication between two groups. Third, drug compliance in the SET group was comparable with that of the TT group. There has been concern about complex administration schedule and higher complication rates of SET than TT [14] which could directly influence on drug compliance and possibly lower drug efficacy. But, our study revealed good compliance of the SET group, almost 85.0%, which was not statistically different from the TT group and showed no significant difference regarding adverse events between the two groups. In our medical center, all physicians explained possible treatment-related complications before prescribing medication with sufficient time, and this was also thought to be the cause of good compliance of SET. Fourth, we suggested reasonable treatment option in cases of treatment failure of first-line SET. One of the major concerns of four-drug regimen is choice of second-line treatment when first-line eradication therapy failed, because there could be more chances of acquiring antibiotic resistance [33]. According to the Maastricht IV Consensus Report, BCQT is recommended as optimal second-line treatment [34], and 2013 revised Korean guidelines also recommends BCQT for second-line option after failure of first-line TT [10]. However, there is no definite guideline for the second-line treatment after failure of SET. According to our results, BCQT could be good second-line treatment option after failure of first-line SET.

This study has several limitations. First, test for *H. pylori* identification or antibiotic sensitivity test was not performed which could clarify direct influence of antibiotic susceptibility on eradication rate. Antibiotic resistance rate, especially clarithromycin resistance is significant factor for determining the efficacy of *H. pylori* eradication with TT or SET [24]. Thus, these kinds of tests are the best way to reduce eradication failure arising from antibiotic resistance [21]. But, it is very difficult to test all patients in the general clinics, and cost-effectiveness is another problem [21]. In a recent prospective study evaluating the efficacy of SET and amoxicillin/tetracycline containing bismuth quadruple therapy (PBAT) for the first-line eradication in the patients from nine different provinces, SET did not reach the 90% eradication rate in the PP analysis despite SET was more effective than PBAT [35]. This discordance with our result could be explained by the difference of local antibiotic resistance. In Korea, it has been reported that antibiotics resistance of *H. pylori* is differ according to the geographic region. In Seoul where our institution is located, resistance rate to clarithromycin is known to be 14.8%, however above study included the provinces in which showed higher resistance rate compared to Seoul such as Busan (42.1%) or Gyeonggi (32.5%) [27], and that might be the cause for the decreased efficacy of SET. The dicision for the appropriate empirical antibiotic therapy should be made based on the data of recentlly updated local antibiotic resistance [11], and therfore nationwide updated data for antibiotic resistance of *H. pylori* should be surveyed.

Second, as this study was conducted retrospectively, there were limitations to obtain detailed medical information such as previous medication history including antibiotics or PPI which could have an influence on eradication failure or diagnosis of *H. pylori* infection. Also, treatment-related adverse events in our study might be down-estimated for the same reason. Compared with previous studies which reported SET-related adverse event rates from 23.3 [14] to 48.0% [32], there was relatively small number of complications in our study (3.3%).

Third, this study enrolled the patients only in the single-center, and the majority of them resided in Seoul. So applying the results of this study to another area could have limitation. However, this study has strength in terms of its large number of study subjects and assessment of the efficacy of rescue therapy after failure of first-line eradication therapy.

## Conclusions

The eradication rate of TT was below the recommended threshold. However, SET showed acceptable eradication rate by both ITT (*P* = 0.001) and PP (*P* = 0.002) analyses with comparable adverse events. SET also has reasonable second-line treatment option, BCQT after failure of first-line SET. Therefore, SET could be effective and tolerable candidate for the first-line *H. pylori* eradication therapy*.*

## Abbreviations

BCQT: Bismuth-containing quadruple therapy
*H. pylori*: *Helicobacter pylori*
ITT: Intention to treat
MALT: Mucosa-associated lymphoid tissue
PP: Per protocol
PPI: Proton-pump inhibitor
SET: Sequential therapy
TT: Triple therapy
vs.: Versus
